# *Trypanosoma cruzi* lineage-specific serology: new rapid tests for resolving clinical and ecological associations

**DOI:** 10.2144/fsoa-2019-0103

**Published:** 2019-10-30

**Authors:** Tapan Bhattacharyya, Niamh Murphy, Michael A Miles

**Affiliations:** 1 Faculty of Infectious & Tropical Diseases, London School of Hygiene & Tropical Medicine, Keppel Street, London WC1E 7HT, UK

**Keywords:** Chagas disease, clinical presentation, genetic diversity, rapid diagnostic tests, serology, sylvatic hosts, *Trypanosoma cruzi*, zoonosis

The protozoan parasite *Trypanosoma cruzi*, the agent of Chagas disease (American trypanosomiasis), remains a major public health concern in Latin America. The insect vectors are haematophagous triatomine bugs that, during or after their blood meal, pass the infective form of *T. cruzi* with feces; the parasite can then enter the host through mucosal membranes, the conjunctiva or abraded skin. Other routes of infection are congenital, oral or via blood/organ donation. The acute phase of infection lasts up to a few weeks, with nonspecific, self-resolving symptoms, although deaths can occur in this phase, particularly in children or young adults. The subsequent chronic phase of infection is life-long unless successfully treated, and asymptomatic (indeterminate) in the majority of patients. However, approximately 30% of infected individuals will develop chronic cardiac and/or gastrointestinal pathologies, with sudden death due to chagasic cardiomyopathy [1]. Chronic symptoms may manifest years or decades after the initial infection; currently, there is no prognostic indicator. A recent WHO report estimates that 5–6 million people are infected with *T. cruzi* [2]. Although vector-borne transmission is confined to the Americas, Chagas disease among migrants from Latin America has become of global health relevance.

*Trypanosoma cruzi* displays remarkable intraspecies diversity, with six genetic lineages (TcI-TcVI) [3], and a proposed seventh, TcBat [4]. The possible association of different lineages of *T. cruzi* with distinct forms of the disease is a long-standing research interest [5]: the cardiac syndrome is found throughout the endemic area, whereas mega syndromes of the colon and esophagus have rarely been reported beyond the southern cone countries of South America.

*Trypanosoma cruzi* is a zoonosis, with all mammals being susceptible to infection, and humans becoming a later host following the historic peopling of the Americas. The association between *T. cruzi* lineages, vectors and domestic, peridomestic and sylvatic animals and ecological cycles is complex [6,7].

Investigating the association between infecting lineage(s) and clinical outcome or ecological cycles has faced significant confounding challenges: the sequestration of the parasite in host tissues during the chronic phase, possibly in a lineage-dependent manner, hampers lineage identification by direct genotyping; *in vitro* culture of isolates may favor the selection of certain lineages. Current serological techniques identify *T. cruzi* specific antibodies (usually IgG), but are not designed to identify infecting lineage. The possibility therefore arose that serology based on lineage-specific *T. cruzi* antigens could overcome these difficulties, as it would allow the identification of an individual's history of lineage infection without the need to genotype or isolate the parasite.

In 2002, Di Noia and colleagues [8] published a pioneering report on TSSA, a mucin expressed on the mammalian bloodstream form of *T. cruzi*. This allowed researchers to enter a new era of lineage-specific serology for this parasite. Following the initial characterization, greater diversity of the protein core of TSSA was revealed [9]. There is a short region of the protein core of *T. cruzi* TSSA where amino acid residues vary according to lineage. Thus, TcI, TcIII and TcIV each have their own potential lineage-specific TSSA epitope; TcII, TcV and TcVI share a common epitope, and the hybrid lineages TcV and TcVI share an additional epitope.

Initially, recombinant TSSA proteins encompassing the TcI or TcII/V/VI common epitopes were produced in *E. coli*, for use in ELISA and western blot. Those reports used sera from mainly southern cone countries, principally Argentina, including those from chronic symptomatic infections [10], pregnant chagasic women [11] and pediatric diagnosis [12]. An alternative approach is to use synthetic peptides (TSSApep-I, -II/V/VI, -III, -IV and -V/VI) representing the lineage-specific epitopes in serological assays. When these were first used in ELISA with sera from a range of South American countries, several novel and unexpected results were found [13]: an association between serological recognition of TSSApep-II/V/VI and degree of clinical symptoms; reaction to TSSApep-II/V/VI was observed in samples from Ecuador and these lineages have rarely been reported in northern South America; specific TSSApep-IV reactions were found in Venezuela and Colombia.

Antibody recognition of the current TSSA-I-specific epitope is rare. This may be due to lack of antigenicity or partially because most assayed sera have originated from regions where TcII, TcV and TcVI are predominant (based on genotyping). This observation stimulated research into the function of the native TSSA isoforms, and assessment of their antigenic properties. Canepa and collegues [14] demonstrated that although the TSSA-II/V/VI isoform had a cell binding and entry capacity, causing the authors to ascribe an ‘adhesin’ function, these properties were lacking in the TSSA-I isoform; a subsequent report also proposed that TSSA-II/V/VI had an additional role in *T. cruzi* differentiation [15]. In terms of antigenicity, both bioinformatic [13] and peptide mapping [16] studies have reinforced the strong antigenicity of the TSSA-II/V/VI common epitope.

The proven efficacy of TSSApep-II/V/VI in ELISA stimulated the development of a lateral flow, immunochromatographic rapid diagnostic test (RDT) called Chagas Sero K-SeT, in which this peptide was immobilized on a nitrocellulose membrane, and specific IgG could be detected by protein G conjugate, within 15 min [17]. This novel *T. cruzi* lineage-specific RDT revealed that in Bolivian patient groups stratified by severity of chagasic cardiomyopathy, RDT seropositivity was five-times higher among patients with severe cardiomyopathy compared with those with no evidence of cardiomyopathy. This was proposed to be due to repeated parasite exposure over time increasing inflammatory cardiac damage in conjunction with an increase in anti-TSSApep-II/V/VI IgG. The same study using Chagas Sero K-SeT also identified sporadic TcII/V/VI infections from Peru.

*Trypanosoma cruzi* has an extremely broad and diverse pattern of circulation among mammals throughout the Americas. Investigation of these natural cycles of infection has led to greatly increased understanding of their natural ecologies, and the transmission risk to human populations. However, the limitations of *T. cruzi* lineage identification described above apply equally to animals and humans. Thus, lineage-specific serology has also been applied to mammals, initially identifying reactions to the TSSA-II/V/VI isoform in Argentine dogs [18]. Serology using the TSSA synthetic peptides has been extended to sylvatic Brazilian primates, identifying *Leontopithecus chrysomelas* (golden-headed lion tamarin) and *L. rosalia* (golden lion tamarin) as natural hosts of TcII and/or TcV/VI in the Atlantic forest of Brazil [19]. The use of Chagas Sero K-SeT with sympatric humans and dogs from northern Argentina has shown the efficacy of protein G for detection across several mammalian orders [20], including primates and rodents (McClean *et al.* Unpublished Observations), thus reducing the need for species-specific secondary antibodies. The use of Protein G and/or Protein A for IgG detection may further extend this range. However, as with human sera, a test for TSSA-I remains elusive. The lack of reactivity to TSSA-I has led to further efforts to identify an alternative robust TcI antigen. Further studies on the TcIII and TcIV epitopes are clearly warranted.

This program of research on *T. cruzi* demonstrates the value and impact of lineage-specific serology. The point-of-care/capture format of the test provides a result in 15 min and is applicable to animals or patients in rural field locations, without access to a laboratory. This enables low cost rapid surveillance for *T. cruzi* lineages among potential animal reservoirs of infection, to assess the risk of emergent endemic regions and to guide control strategies, without the need to isolate *T. cruzi* from the animals. Furthermore, we can far more efficiently investigate whether the genetically distinct *T. cruzi* lineages may be responsible for the different clinical presentations and prognoses of Chagas disease. This approach also clearly has potential wider application to other infectious diseases.
